# Recipient Outcomes after ABO-Incompatible Liver Transplantation: A Systematic Review and Meta-Analysis

**DOI:** 10.1371/journal.pone.0016521

**Published:** 2011-01-25

**Authors:** Jian Wu, SunYi Ye, XiaoFeng Xu, Haiyang Xie, Lin Zhou, ShuSen Zheng

**Affiliations:** 1 Key Laboratory of Combined Multi-Organ Transplantation, Ministry of Public Health, First Affiliated Hospital, School of Medicine, Zhejiang University, Hangzhou, China; 2 Key Laboratory of Organ Transplantation, First Affiliated Hospital, School of Medicine, Zhejiang University, Hangzhou, China; 3 Division of Hepatobiliary and Pancreatic Surgery, Department of Surgery, First Affiliated Hospital, School of Medicine, Zhejiang University, Hangzhou, China; University of California Los Angeles, United States

## Abstract

**Background:**

ABO-incompatible live transplantation (ILT) is not occasionally performed due to a relative high risk of graft failure. Knowledge of both graft and patient survival rate after ILT is essential for donor selection and therapeutic strategy. We systematically reviewed studies containing outcomes after ILT compared to that after ABO-compatible liver transplantation (CLT).

**Methodology/Principal Findings:**

We carried out a comprehensive search strategy on MEDLINE (1966–July 2010), EMBASE (1980–July 2010), Biosis Preview (1969–July 2010), Science Citation Index (1981–July 2010), Cochrane Database of Systematic Reviews (Cochrane Library, issue 7, 2010) and the National Institute of Health (July 2010). Two reviewers independently assessed the quality of each study and abstracted outcome data. Fourteen eligible studies were included which came from various medical centers all over the world. Meta-analysis results showed that no significantly statistical difference was found in pediatric graft survival rate, pediatric and adult patient survival rate between ILT and CLT group. In adult subgroup, the graft survival rate after ILT was significantly lower than that after CLT. The value of totally pooled OR was 0.64 (0.55, 0.74), 0.92 (0.62, 1.38) for graft survival rate and patient survival rate respectively. The whole complication incidence (including acute rejection and biliary complication) after ILT was higher than that after CLT, as the value of totally pooled OR was 3.02 (1.33, 6.85). Similarly, in acute rejection subgroup, the value of OR was 2.02 (1.01, 4.02). However, it was 4.08 (0.90, 18.51) in biliary complication subgroup.

**Conclusions/Significance:**

In our view, pediatric ILT has not been a contraindication anymore due to a similar graft and patient survival rate between ILT and CLT group. Though adult graft survival rate is not so satisfactory, ILT is undoubtedly life-saving under exigent condition. Most studies included in our analysis are observational researches. Larger scale of researches and Randomized-Control Studies are still needed.

## Introduction

ABO-incompatible liver transplantation was regarded as a relative contraindication because of high incidence of bile duct and vascular complications. In the early stage, the graft failure rate was unacceptably 50% due to humoral rejection [1], [2], [3], [4]. Currently, there is a great shortage of liver donors all over the world. The number of patients in the waiting list is always multiple times of the number of liver donors. To lessen humoral rejection, a variety of strategies have been tried, including plasmapheresis, hepatic perfusion, various immunosuppressive agents, steroids, Rituximab and splenectomy [5], [6]. Particularly in recent years, advanced new immunosuppressive agents are developed, patient and graft survival rate after ILT has increased dramatically. However, high risk of complication after ILT, such as biliary complication[7], acute rejection[6], [8], [9] and hepatic artery thrombosis, is still a vital issue related to ILT.

To our knowledge, no systematic evaluation has been performed on graft/patient survival rate or complication incidence. The objective of this study was to summarize different outcomes between ILT and CLT group. Clarifying the graft/patient survival rate and complication incidence between two groups may give a new protocol for liver transplantation when different ABO blood type is taken into account.

## Methods

### Ethics

All included data were from the published literature and there was no ethical approval required.

### Searching

To identify published and unpublished reports of relevant studies, we searched relevant electronic databases, including MEDLINE (1966–July 2010), EMBASE (1980–July 2010), Biosis Preview (1969–July 2010), Science Citation Index (1981–July 2010), Cochrane Database of Systematic Reviews (Cochrane Library, issue 7, 2010).We also searched for unpublished trials and those in progress using repositories of clinical trials, including the National Institute of Health (July 2010). Google Scholar was also used to find fulltexts. The websites of European Liver Transplant Registry (ELTR), Scientific Registry of Transplant Recipients (SRTR) and China Liver Transplant Registry (CLTR) were researched for additional information. Searches were not restricted by year of publication in Chinese or English. The search strategy included the terms “ABO* AND liver transplantation*”. Reference lists of all studies included were scanned to identify additional potentially relevant studies. Two reviewers independently screened the titles and abstracts of identified papers, and most potentially relevant studies' full text copies were obtained.

ABO-incompatible liver transplantation include the following donor to recipient pairings: A to B,O; B to A,O; AB to A,B,O. Other pairings (including identical pairings) are considered ABO-compatible. Biliary complications include biliary leakage and stricture after liver transplantation. Clinical rejection is defined as an increase in the liver enzymes with corresponding liver biopsy or response to increased drug dose (immunosuppressive or steroids) without liver biopsy.

### Selection

We conducted this systematic review and meta-analysis according to a prespecified protocol, which was guided by the Meta-analysis of Observational Studies in Epidemiology consensus statement [10] and the PRISMA Statement [11]. Those studies which had a comparison between ILT and CLT group in graft survival rate, patient survival rate or complication incidence met our criteria.

### Validity assessment

Because of the ethic limitations, it is hard to perform a randomized controlled trial on this topic. All of the studies included in this research were observational studies.

### Data Abstraction

Two reviewers(SYY,XFX)independently evaluated each citation and abstracted information, disagreements were resolved by discussion. Another review (JW) independently confirmed the accuracy of all abstracted data. To quantify the level of agreement between reviewers, a κ statistic was calculated. The κ statistic is a chance-corrected proportional index, with values ranging from +1 (perfect agreement) to −1 (complete disagreement).The mean value of κ fell into the range of 0.75–1.00 was viewed as high agreement. For all included studies, we abstract the following data from original publications: first author and year of publication; country; target population; study size; 1-,3-,5-,10-year graft survival rate and patient survival rate; acute rejection incidence and biliary complication incidence.

### Statistical Analysis

We drew information from each accordant article. If necessary data was not showed directly in the article, we attempted to contact authors to get original data (but failed to get useful information), and a data extraction software-Engauge Digitizer (Free Software Foundation, Boston, US) was also used to get details. The meta-analysis was done consists with recommendations from the Cochrane Collaboration and the PRISMA Statement [11] with standard software (Revman 5.0 and Stata version 10.0).

### Quantitative data synthesis

Heterogeneity was assessed with I^2^ statistics [12]. I^2^ is the proportion of total variation across studies that is due to heterogeneity rather than chance (sampling error). I^2^ values of 25%, 50% and 75% respectively represents the cut off line of low, moderate and high heterogeneity [13].Odds ratio (OR) was used as the summary statistic to perform statistical analysis of dichotomous variables. A fixed-effect model was used for calculations of summary estimates and their 95% CI. However, when the heterogeneity was high (I^2^>50%), a random-effects model was used. A p value of less than 0.05 was considered to indicate statistically significant difference. Sensitivity analysis was performed using the method of excluding extreme data (the maximum or the minimum). We used Egger test (Stata version 10.0) to examine the potential risk of publication bias. Publication bias was indicated when p value was less than 0.10.

## Results

### Flow of included studies

**Figure 1:**
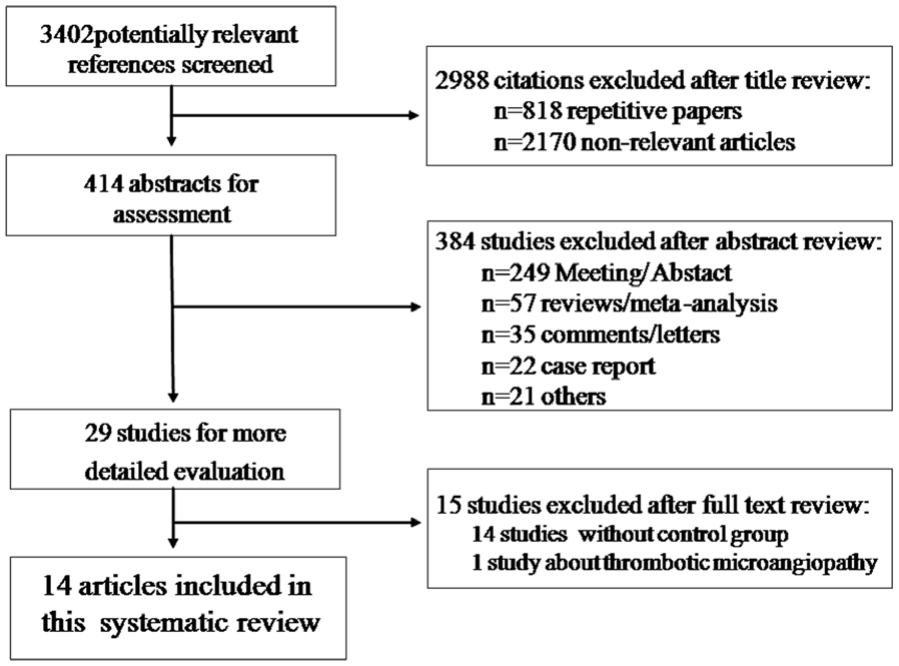
Flow of included studies.

We screened 3401 citations, from which 413 abstracts are used for further assessment (Fig. 1). Twenty-nine articles met our criteria. Sixteen were subsequently excluded because they did not contain the detail what we exactly need. Thus, we finally identified fourteen articles to assess patient/graft survival rate as well as incidence of various complications after ILT. However, they were all observational studies without random or control. After adjustment for chance concerning the agreement between reviewers, the κ coefficient on the agreement of the included studies was 0.83 (0.95 CI 0.77–0.89), showing good agreement between reviewers in data extraction.

### Study Characteristics

The 14 [1], [2], [8], [14], [15], [16], [17], [18], [19], [20], [21], [22], [23], [24] studies were published from 1990 to 2009. The characteristics are presented in Table 1. Thirteen were English and 1 was Chinese (Yang et al.).The studies were from 10 countries: 5 studies from the America, 3 from Japan, 1 from Canada, 1 from Australia, 1 from China, 1 from Belgium, 1 from France and 1 study from Nordic countries (Norway, Sweden, Denmark, Finland). All the results of Meta-analysis were showed in Table 2.

**Table 1:** Characteristics of included studies.

Study	Country	Publication	population	No. of ILT/CLT
Yang (14)	China	2007	All ages	21/45
Toso (15)	Canada	2007	Adult	14/94
Ueda (16)	Japan	2006	Pediatric	74/494
Heffron (17)	US	2010	Pediatric	12/21
Heffron (8)	US	2006	Pediatric	16/122
Chui (18)	Australia	1997	All ages	7/36
Cacciarelli (19)	US	1995	Pediatric	14/130
Sanchez (20)	US	1993	All ages	7/36
Reding (21)	Belgium	1992	All ages	16/54
BJØRO (22)	Nordic	2003	Adult	10/219
Tokunaga.(23)	Japan	1993	Pediatric	3/31
Iwamoto (24)	Japan	2008	Adult	15/37
Gugenheim (2)	France	1990	All ages	17/217
Stewart (1)	US	2009	Infant	130/390
			Pediatric	116/348
			Adult	585/1755

**Table 2:** Meta-analysis results.

Outcomes	Number of studies	Number of participants		Test of homogeneity		OR(95% CI)	P value
		CLT	ILT	I^2^(%)	P value		
Graft survival							
1-y All ages	5	2818	889	74	0.004	0.30(0.12,0.75)	0.00
Pediatric	4	1094	220	0.0	0.46	0.83(0.59,1.17)	0.28
Adult	3	2601	872	0.0	0.76	0.60(0.50,0.73)	0.00
3-y Pediatric	3	506	146	0.0	0.81	0.96(0.64,1.43)	0.84
Adult	2	1974	695	35	0.22	0.46(0.38,0.55)	0.00
5-y Pediatric	2	842	190	9 9	0.29	0.75(0.53,1.06)	0.10
Adult	3	2068	609	0.0	0.43	0.68(0.56,0.81)	0.00
10-y Pediatric	2	842	190	67	0.08	0.73(0.39,1.25)^a^	0.06^a^
Total(95%CI)		12016	3531	39	0.08	0.73(0.39,1.25)^a^	0.00 a
Patient survival							
1-y	7	872	94	13	0.33	0.87(0.52,1.46)^a^	0.59^a^
3-y	2	349	24	0.0	0.80	0.62(0.24,1.59)	0.32
5-y	2	459	24	0.0	0.38	1.52(0.61,3.79)	0.37
Total(95%CI)		1463	125	0.0	0.53	1.02(0.66,1.58)	0.93
Complications							
Biliary complication	4	222	70	80	0.002	0.35(0.06,2.14)^a^	0.25 a
Acute rejection	4	216	58	53	0.09	1.23(0.38,4.04)^a^	0.76 a
Total(95%CI)		438	128	73	0.00	0.64(0.22,1.89)^a^	0.42 a

a Random-effects model.

### Graft and Patient Survival rate

**Figure 2:**
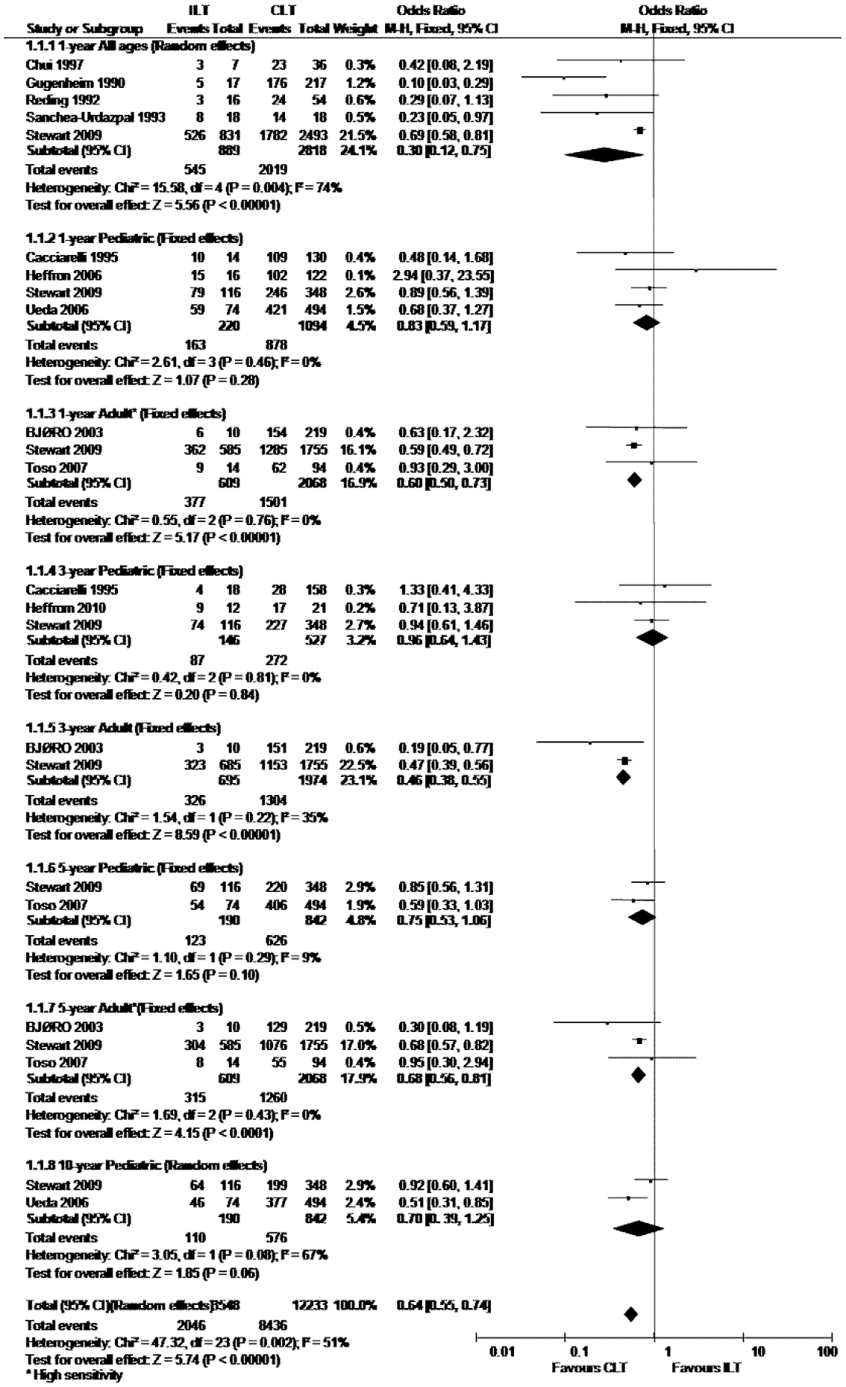
Meta-analysis results of graft survival rate.

**Figure 3:**
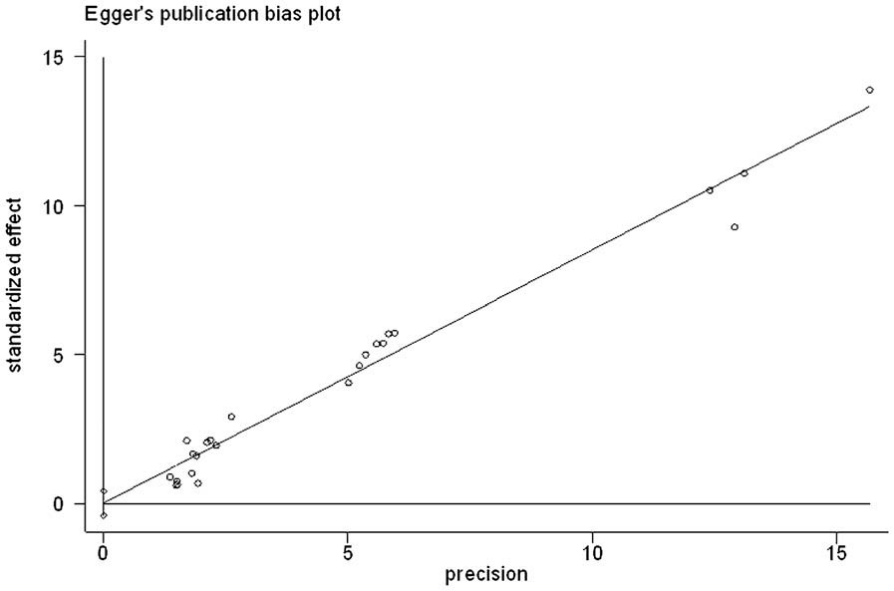
Egger test results of studies on graft survival rate.

**Figure 4:**
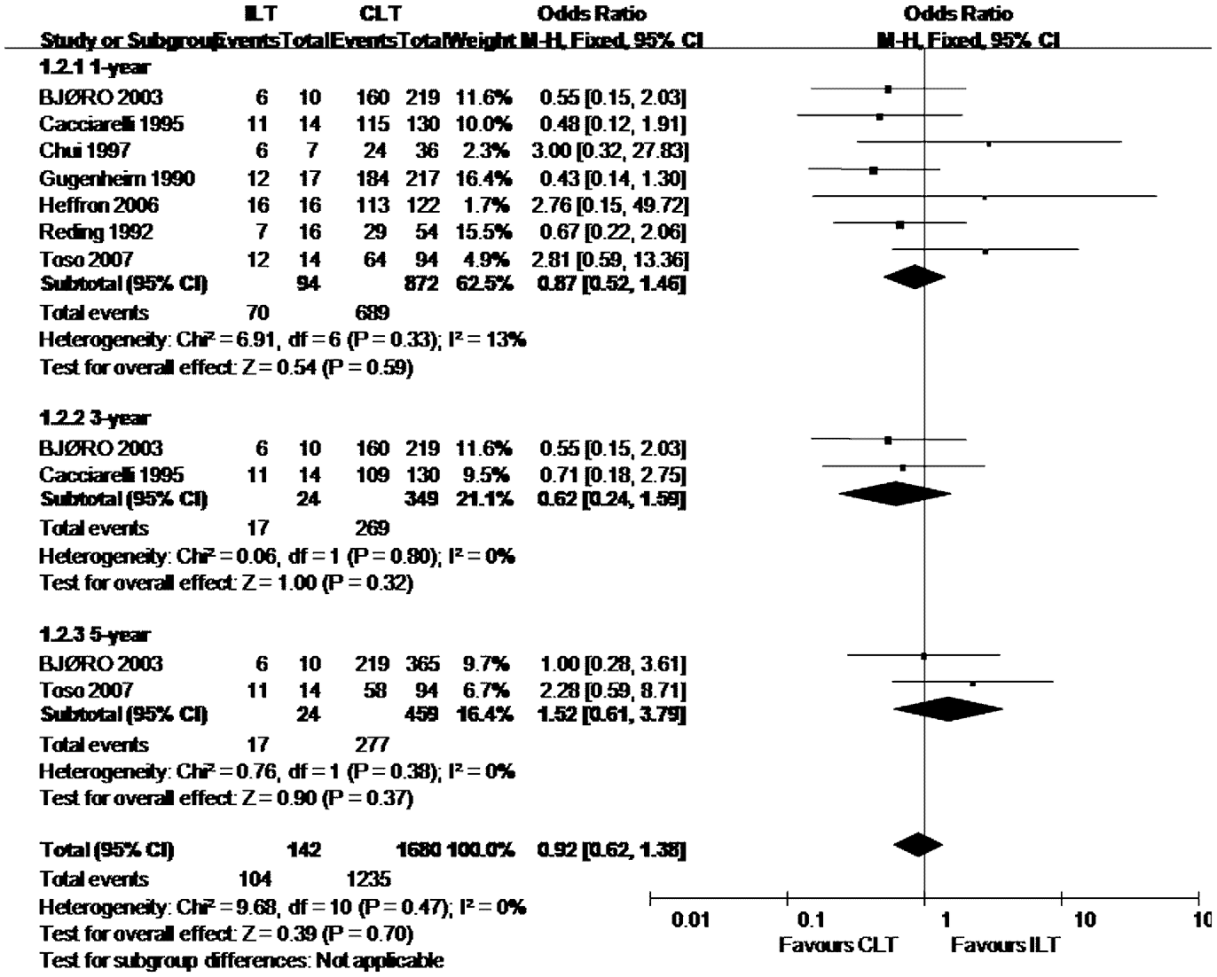
Meta-analysis results of patient survival rate.

**Figure 5:**
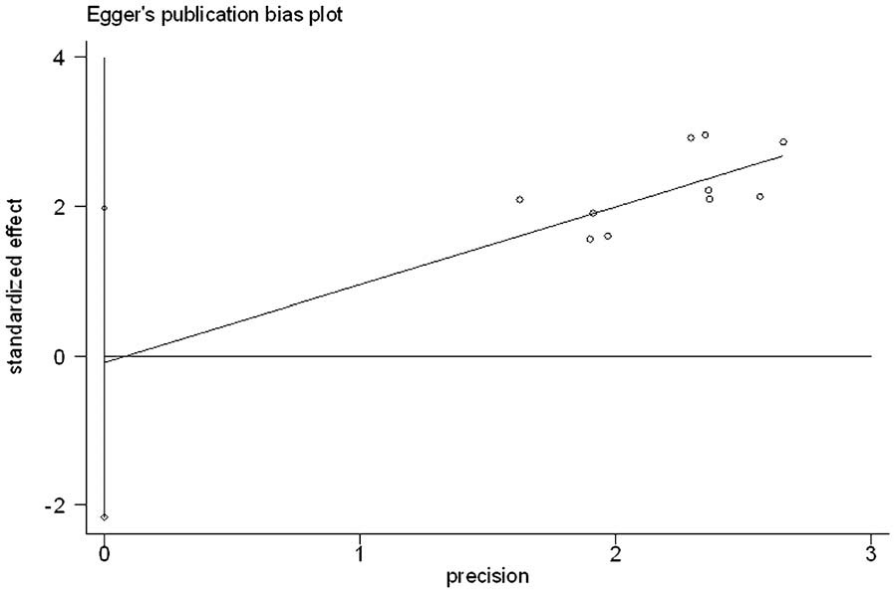
Egger test results of studies on patient survival rate.

We analyzed the graft survival rate between ILT group and CLT group according to different age range. The totally pooled OR was 0.64 (0.55, 0.74) (Random-effects model). This result was statistically significant (p<0.05) (Fig. 2). Egger test did not indicate obvious publication bias (p = 0.904) (Fig. 3). Further more, patient survival rate was also collected for analysis. The totally pooled OR was 0.92 (0.62, 1.38) (Fig. 4). This result was not statistically significant (p = 0.70) (Random-effects model). Egger test did not indicate obvious publication bias (p = 0.925) (Fig. 5).

### One-year graft survival rate

There were 5 studies contained the 1-year graft survival rate between two groups including all aged cases. Among the 5 articles, the study of Stewart et al. contained the most cases, in which 831 patients received ABO-incompatible liver transplantation, and there were 2818 patients accepted ABO-compatible liver transplantation as controlled group. The study size of other 4 articles were much smaller, they totally contained 43, 234, 70 and 36 cases respectively. The test of homogeneity showed that results were heterogeneous across studies (p = 0.004, I^2^ = 74%) and a random-effects model was used. Meta-analysis showed the pooled OR was 0.30 (0.12, 0.75). This result was statistically significant (p<0.05). Sensitivity analysis was also performed, it owned a low sensitivity.

The result of pediatric 1-year graft survival rate is based on another 4 studies: the study of Stewart et al., Ueda et al., Heffron et al., and Cacciarelli et al.. The study size was respectively 464, 568, 138, and 144. This group of study size was much more balanced. The test of homogeneity showed that results were coherent across studies (p = 0.46, I^2^ = 0.0%) and a fixed-effects model was used. Meta-analysis showed the pooled OR was 0.83 (0.59,1.17). This result was not statistically significant (p = 0.28). A low sensitivity was presented after performing sensitivity analysis.

There were 3 studies contained the information about adult 1-year graft survival rate. They were study of Stewart et al., Toso et al. and Cacciarelli et al.. Their study size was 2340, 108 and 229 respectively. The test of homogeneity showed that results were coherent across studies (p = 0.76, I^2^ = 0.0%). So we used a fixed-effects model. Meta-analysis showed the pooled OR was 0.6 (0.50, 0.73). This result was statistically significant (p<0.05). However, this subgroup showed a high sensitivity after performing sensitivity analysis.

### One-year patient survival rate

The result of patient 1-year survival rate was based on 7 studies. They were Heffron et al., Toso et al., Chui et al., Reding et al., BJØRO et al., Gugenheim et al. and Caccuarelli et al.. Their study size swayed from 43–234. The test of homogeneity showed that results were coherent across studies (p = 0.33, I^2^ = 13%). Meta-analysis showed the pooled OR was 0.87 (0.52, 1.46). This result was not statistically significant (p = 0.59). A low sensitivity was presented after performing sensitivity analysis.

### Three-year graft survival rate

There were 3 studies provided 3-year graft survival rate on pediatric liver transplantation between ILT and CLT groups. They were Heffron et al., Stewart et al., and Cacciarelli et al., and their study size were 17, 227 and 28 respectively. The test of homogeneity showed that results were coherent across studies (p = 0.81, I^2^ = 0.0%). Meta-analysis showed the pooled OR was 0.96 (0.64, 1.43). This result was not statistically significant (p = 0.84). A low sensitivity was presented after performing sensitivity analysis.

The result of graft 3-year survival rate about adult was based on 2 studies. They were Stewart et al., BJØRO et al., and their study size were 1153 and 151. The test of homogeneity showed that results were coherent across studies (p = 0.22, I^2^ = 35%). Meta-analysis showed the pooled OR was 0.46 (0.38, 0.55). This result was statistically significant (p<0.05). A low sensitivity was presented after performing sensitivity analysis.

### Three-year patient survival rate

The result of patient 3-year survival rate was based on 2 studies. They were BJØRO et al., and Caccuarelli et al., and their study size were 160 and 109. The test of homogeneity showed that results were coherent across studies (p = 0.8, I^2^ = 0.0%). Meta-analysis showed the pooled OR was 0.62 (0.24, 1.59). This result was not statistically significant (p = 0.32). A low sensitivity was showed.

### Five-year graft survival rate

There were 2 studies provided 5-year graft survival rate on pediatric liver transplantation between ILT and CLT groups. They were Stewart et al., and Toso et al., and their study size were respectively 220 and 406. The test of homogeneity showed that results were coherent across studies (p = 0.29, I^2^ = 9%). Meta-analysis showed the pooled OR was 0.75 (0.53, 1.06). This result was not statistically significant (p = 0.1). A low sensitivity was presented.

The result of graft 5-year survival rate about adult was based on 3 studies. They were Stewart et al., Toso et al., and BJØRO et al., and their study size were 1076, 55, and 129. The test of homogeneity showed that results were coherent across studies (p = 0.43, I^2^ = 0.0%). We used a random-effects model. Meta-analysis showed the pooled OR was 0.68 (0.56, 0.81). This result was statistically significant (p<0.05). However, a relatively high sensitivity was showed in this subgroup.

### Five-year patient survival rate

The result of patient 5-year survival rate was based on 3 studies. They were Toso et al.,and BJØRO et al., and their study size were 58, and 219. The test of homogeneity showed that results were coherent among the included studies. We used a fixed-effects model. Meta-analysis showed the pooled OR was 1.52 (0.61, 3.79). This result was not statistically significant (p = 0.37) and showed a low sensitivity.

### Ten-year graft survival rate

The result of graft 10-year survival rate on pediatric was based on 2 studies. The study of Stewart et al., and Ueda et al., and their study size were 199 and 377. The test of homogeneity showed that results were heterogeneous among the included studies. We used a random-effects model. Meta-analysis showed the pooled OR was 0.73 (0.39, 1.25). This result was not statistically significant (p = 0.06). The adult 10-year graft survival rate was based on only 1 study.

### Complications

**Figure 6:**
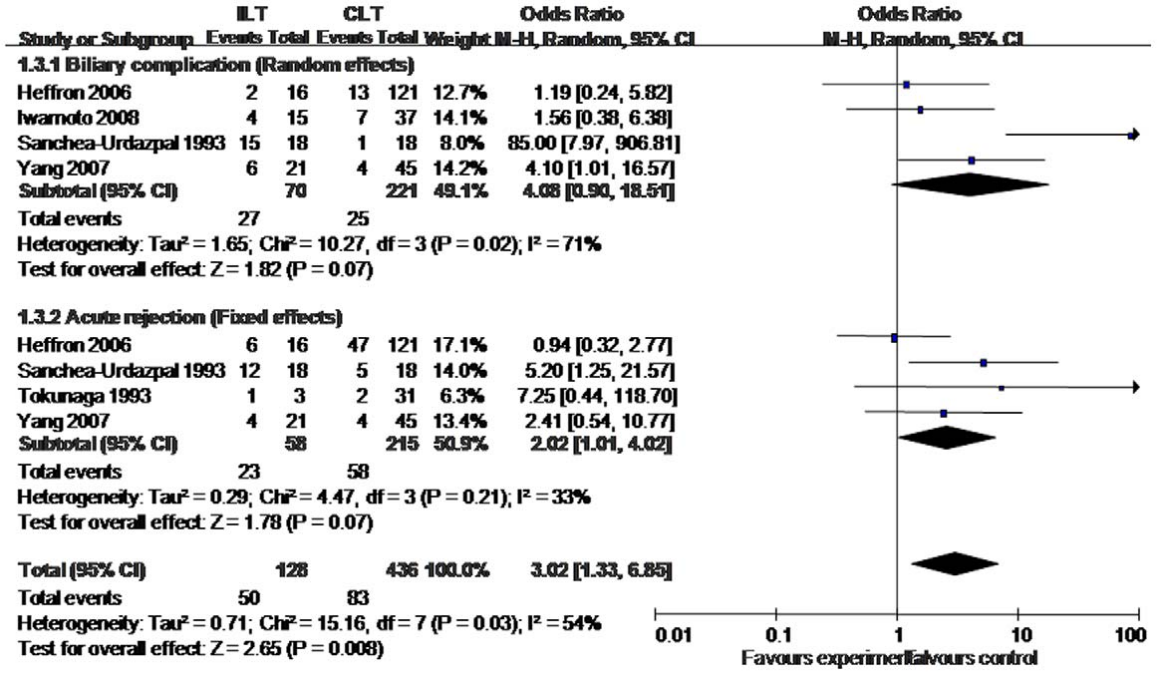
Meta-analysis results of complication incidence.

**Figure 7:**
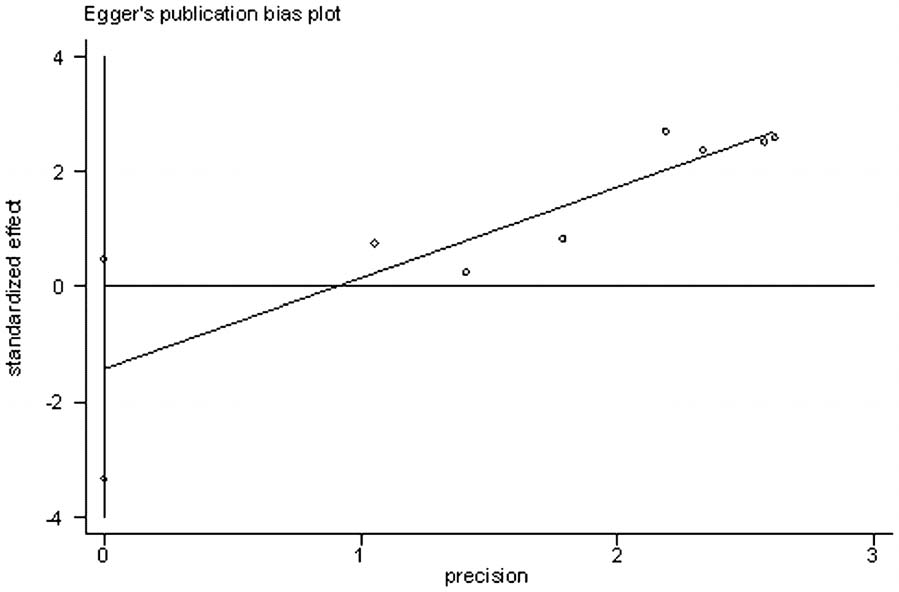
Egger test results of studies on complication incidence.

Several complications after liver transplantation were searched and evaluated, we finally selected acute rejection and biliary complication for analysis. We used a random-effects model for meta analysis. The totally pooled OR of complication was 3.02 (1.33, 6.85) (Fig. 6), which suggested that the whole complication incidence after ILT was higher than that after CLT. This result was statistically significant (p = 0.018). Sensitivity analysis and Egger test (Fig. 7) were also performed. No publication bias was observed (p = 0.711).

### Biliary complication

The result of biliary complication incidence was based on 4 studies (Fig. 6). The study of Iwamoto et al., Yang et al., Heffron et al., and Sanchez-Urdazpal et al., and their study size were 52, 66, 137, and 36. The test of homogeneity showed that results were heterogeneous among the included studies. We used a random-effects model. Meta-analysis showed the pooled OR was 4.08 (0.90, 18.51). A low sensitivity was presented after performing sensitivity analysis.

### Acute rejection

There were also 4 studies included in this group for analysis (Fig. 6). The study of Yang et al., Heffron et al., Tokunaga et al., and Sanchez-Urdazpal et al., and their study size were 66, 137, 34, and 36. The test of homogeneity showed that results were homogeneous among the included studies. We used a fixed-effects model. Meta-analysis showed the pooled OR was 2.02 (1.01, 4.02). A low sensitivity was presented after performing sensitivity analysis.

## Discussion

We comprehensively reviewed the literature on survival rate and complication outcomes of ABO-incompatible liver transplantation. Analysis was mainly performed in pediatric and adult subgroups, infant subgroup analysis was not included in our research due to insufficient data. Our meta-analysis results showed that there was no statistically significant difference in pediatric graft survival rate no matter 1-year, 3-year, 5-year, and 10-year graft survival rate. However, the 1-year, 3-year, and 5-year graft survival rate on adult had statistical difference between ILT and CLT group. The graft survival rate of CLT group surpassed that of ILT group. The 1-year, 3-year, and 5-year patient survival rate was not statistically different. The patient survival rate after ILT was elevated mainly by retransplantation. When it comes to complication, no statistical difference was demonstrated by our results on neither acute rejection nor biliary complication. ABO-incompatible liver transplantation might not be a high risk factor of complication after liver transplantation.

ABO-incompatible liver transplantation was considered inappropriate because of its theoretically high risk of humoral rejection. Early efforts showed that organ loss and patient death with graft failure rates from 30% to more than 50%, and nearly half of the ILT patients ultimately requiring retransplantation. According to previous studies, age played an important role in the development of allograft failure [25], [26]and younger recipients turned better outcomes after ILT [27]. Egawa et al. indicated that the patient survival rate after ILT gradually decreased with the rise of recipients' age. The 5-year patient survival rate was 85% in infants and only 52% in adults[28]. Moreover, Heffron demonstrated that the 1-year actual graft survival rate (92.3%) after ILT is higher than that after CLT (83.4%) utilizing standard immunosuppression with selective postoperative plasmapheresis[8]. Our meta-analysis results confirmed that pediatric graft survival rate after ILT liver transplantation was not obviously different from that of CLT group though we did not analyze the detail for infant. On the other hand, adult graft survival rate after ILT was much lower. Several reasons may accounted for this phenomenon: first, anti-A and anti-B antibody titers remain at low levels at early age because of its incomplete capacity of immune system [29]. Second, the complement system was not so sensitive compared to adults [30]. Recipients' age plays an important role in prognosis after liver transplantation. According to the data of ELTR, children's survival rate was much higher than adults'. Similarly, the result of ABO-incompatible liver transplantation in adult seemed more serious. Our results showed that the graft survival rate of adult after ILT was obviously lower than after CLT.

Acute rejection, biliary complication as well as infection mainly accounted for graft failure after ILT. Antibodies that against the donor-blood group antigens bound to the graft vascular endothelium, inducing complement fixation, endothelial damage and the formation of platelet thrombi which followed by the clotting cascade, causing hemorrhagic necrosis. In early failed ILT grafts, pathologic examination showed widespread areas of geographic hemorrhagic necrosis, antibody and complement components were deposited in arteriole [31]. Except antibody-mediated rejection, acute rejection also contains T cell-mediated rejection. They two always appear at the same time when acute rejection occurs [32]. The graft had been assumed to be resistant to antibody-mediated rejection in ABO-compatible liver transplantation[33],we believe that antibody-mediated rejection plays a more important role in ILT than in CLT. Immunological lesions in arteriole always lead to lethal biliary complication such as ischemic cholangitis, also called ischemic-type biliary lesion (ITBL). The dominant feature of ischemic cholangitis is biliary stricture[34]. Most ischemic stricture patients need retransplantation [35].

According to the study of Sanchez-Urdazpal, biliary complication and rejection incidence after ILT was much higher than that after CLT [20]. Our results showed that the whole complication incidence and acute rejection incidence after ILT were higher than that after CLT. Though biliary complication incidence after ILT was on the increase compared to that after CLT, it did not show statistical difference since its OR scope contained 1 (0.90, 18.51). One explanation is that the number of studies included in biliary complication subgroup was relatively small. Another factor is that authors were reluctant to report their high complication incidence results, and willing to share their successful experience. Several reports indicated that administration of rituximab and plasmapheresis before transplantation can reduce the incidence of antibody-mediated rejection both in DDLT and LDLT[36], [37].Using the therapeutic regimen of perioperative plasmapheresis, intrahepatic arterial infusion, splenectomy, and triple- or quadruple-drug therapy containing calcineurin inhibitor, steroid, and cyclophosphamide, azathioprine, or MMF, the survival rate after ILT has been promoted greatly. Hanto reported that there was no immunological graft loss using total plasma exchange, splenectomy, and quadruple immunosuppression [6], [38], [39], [40].

### Limitations

To our knowledge, this is the first comprehensive review of this topic. We believe our search strategy was sufficient and included all relevant articles. Several reviewers attended to identify all these articles and we used subgroup analysis, which minimized potential selection biases and ensured accuracy of the abstracted data. Our systematic review has several limitations. First, there was no randomized studies on our topic, all of them were observational studies. And there was only one article clearly stated its match-control method in collecting its original data. Second, the number of included studies and participants in each subgroup analysis was relatively small. Third, some subgroups (1-year adult graft survival rate, 5-year adult graft survival rate, 5-year patient survival rate) had relatively high sensitivity, which was mainly caused by the relatively larger study size from the study of Stewart. It was a national registry analysis from the United States. However, we failed to get similar registry reports from other Europe or Asia. Otherwise, the analysis result might be more comprehensive and representative. The meta-analysis results of these three subgroups should be carefully concluded. Fortunately, it did not affect the pooled results of total graft survival rate and patient survival rate. In order to get convinced results, more large scale of statistical data and Randomized-Control Study should be needed. Fourth, we did a mixed analysis and did not differentiate LDLT or DDLT. Because most studies only had mixed results and the information in each group was insufficient for analysis. Fifth, potential bias has several considerations: included studies were non-randomized researches; the study sizes were relatively small; the relatively high heterogeneity among studies; some subgroups included only a few studies; chance related bias.

In conclusion, ABO-incompatible liver transplantation is still an inferior selection due to its relatively low graft survival rate though therapeutic strategy for ILT has been improved recently. ILT in pediatric is feasible because patient/graft survival rate is no obviously difference compared to CLT. Pediatric ILT has not been a contraindication anymore. Though adult graft survival rate is not so satisfactory, ILT is undoubtedly being viewed as a vital option for patients with acute liver failure requiring exigent liver transplantation. In addition, adult patient survival rate and complication incidence are still acceptable due to retransplantation. However, some larger scale of researches and Randomized-Control Studies are still needed on ABO-incompatible liver transplantation.
